# Effect of Collagen Hydrolysate and Fish Oil on High-Sensitivity C-Reactive Protein and Glucose Homeostasis in Patients with severe Burn; a Randomized Clinical Trial

**DOI:** 10.22037/aaem.v9i1.1289

**Published:** 2021-07-05

**Authors:** Elham Alipoor, Mohammad Javad Hosseinzadeh-Attar, Shiva Salehi, Mostafa Dahmardehei, Mehdi Yaseri, Mohammad Reza Emami, Mehdi Hajian, Seyed Mahdi Rezayat, Shima Jazayeri

**Affiliations:** 1 Department of Nutrition, School of Public Health, Iran University of Medical Sciences, Tehran, Iran.; 2 Department of Clinical Nutrition, School of Nutritional Sciences and Dietetics, Tehran University of Medical Sciences, Tehran, Iran.; 3 Cardiac Primary Prevention Research Center, Cardiovascular Diseases Research Institute, Tehran University of Medical Sciences, Tehran, Iran.; 4 Department of Plastic Surgery, Burn Research Center, Motahari Hospital, Iran University of Medical Sciences, Tehran, Iran.; 5 Department of Epidemiology and Biostatistics, School of Public Health, Tehran University of Medical Sciences, Tehran, Iran.; 6 Motahari Hospital, Iran University of Medical Sciences, Tehran, Iran.; 7 Department of Pharmacology, School of Medicine, Tehran University of Medical Sciences, Tehran, Iran.; 8 Department of Nanomedicine, School of Advanced Technologies in Medicine, Tehran University of Medical Sciences, Tehran, Iran.; 9 Research Center for Prevention of Cardiovascular Disease, Institute of Endocrinology and Metabolism, Iran University of Medical Sciences, Tehran, Iran.

**Keywords:** Burns, Collagen, Inflammation, Insulin resistance, Fatty Acids, Omega-3

## Abstract

**Introduction:**

Collagen and omega-3 fatty acids (FAs) are suggested to have anti-inflammatory, anti-oxidant, and insulin-sensitizing properties. The aim of this study was to investigate the effect of collagen hydrolysate and omega-3 FAs on inflammation and insulin resistance in patients with major burns.

**Methods:**

In this double-blind randomized clinical trial, 66 patients with 20-45% burns were assigned to either of the three groups of collagen (40 gr/d), collagen (40 gr/d) plus fish oil (10 ml/d), or control. High-sensitivity C-reactive protein (hs-CRP), fasting blood glucose (FBG) and insulin concentrations, and homeostatic model assessment for insulin resistance (HOMA-IR) were assessed at baseline, as well as end of weeks two and three.

**Results:**

Based on post-hoc analyses, hs-CRP levels were significantly lower in the collagen (p=0.026) and collagen+omega-3 (p=0.044) groups compared to the control group, at week three. However, pre- to post- (week three) changes of hs-CRP were significantly higher only in the collagen+omega-3 group compared to the control group (173.2 vs. 103.7 mg/l, p=0.024). After three weeks of the intervention, insulin (11.3 and 11.9 vs. 22.8 µIU/ml) and HOMA-IR (2.9 and 2.8 vs. 7.9) values seemed to be clinically, but not statistically, lower in both intervention groups compared to the control group. Pre- to post- (week three) values of FBG decreased significantly in the collagen (p=0.002) and collagen+omega-3 (p=0.036) groups. Insulin (p=0.008) and HOMA-IR (p=0.001) decreased significantly only in the collagen+omega-3 group at week three compared to the baseline.

**Conclusions:**

Supplementation with collagen hydrolysate and omega-3 FAs can improve hs-CRP concentration and probably insulin resistance in patients with severe burns. Omega-3 FAs had additional effects on modulating inflammation. Larger clinical trials are needed to confirm the current findings especially in terms of glucose homeostasis.

## 1. Introduction

Major burns are recognized among the most severe physiologic stresses, which are accompanied with a specific metabolic response (1). Burns are among the most prevalent types of injuries (2), which impose high costs on the healthcare systems proportional to the severity of the condition (3). Despite significant reduction in post-burn mortality over the recent decades, morbidities are still a serious clinical challenge (4). Hypermetabolism is a pivotal pathophysiologic response in burns (1, 4), which is associated with other profound disorders including inflammation, oxidative stress, and insulin resistance (4-6). Inflammatory mediators are major players in hypermetabolic response, which increase considerably after burn injury and may last for weeks depending on the severity of injury (5). Glucose metabolism is remarkably altered in thermal injuries and insulin resistance may be prolonged following burns (1, 4). Endoplasmic stress in muscles, skin, and adipose tissue are correlated with structural changes in insulin receptors and mitochondrial dysfunction, which in turn lead to glucose intolerance and disturbed lipid metabolism (7, 8). Catecholamines and stress hormones, especially cortisol, are other main mediators of the hypercatabolic response (1, 4), which increase glucose flow through induction of proteolysis, lipolysis, and gluconeogenesis, and also trigger systemic inflammation (1). High glucose production is also largely associated with burn wounds to support the glucose demand of cells during anaerobic metabolism (9, 10). The main aim of such systemic endocrine and metabolic responses in metabolic stresses are to support normal homeostasis and prevent complications such as infection. However, certain aspects of this response are maladaptive and in the case of glucose intolerance it may lead to delayed wound healing, high risk of sepsis, organ failure and mortality (8, 11). Prolonged inflammation may also delay wound healing (12). Thus, effective interventions are required to modulate post-burn metabolic disorders. 

Nutritional therapy is necessary in patients with major burns to provide metabolic needs as well as to modulate hypermetabolism and its consequences (13). Providing adequate amounts of high-quality proteins is of great importance due to heavy losses of protein, increased gluconeogenesis, and tissue repair (14). Collagen is a protein source, which has received an increasing attention recently. The beneficial effects of collagen have been suggested on wound healing and tissue repair in burns (15), diabetes (16) and aging (17). It has been suggested that collagen also has anti-inflammatory, anti-oxidant, and anti-diabetic properties (18-20) in excisional wounds and other experimental models, which have not yet been investigated in burns. Omega-3 fatty acids (FAs) are other important nutrients with anti-inflammatory properties, which were reported to be advantageous in reduction of infection, sepsis, and septic shock (21, 22), as well as decreasing hospital (21) and intensive care unit (23) stay in burns. Studies in burns have mainly investigated the anti-inflammatory effects of omega-3 FAs in combination with other nutrients as enteral or parenteral formulas (21, 23, 24) or from plant sources (25), and thus the reported results are conflicting. Omega-3 FAs could also improve insulin resistance (26), which has been limitedly investigated in burns. Thus, considering the importance of alleviating post-burn pathophysiological disorders with appropriate interventions and given the promising effects reported for collagen and omega-3 FAs in non-burn studies, this trial aimed to assess the effect of collagen hydrolysate and omega-3 FAs on inflammation and glucose homeostasis in patients with major burns.

## 2. Methods

***2.1. Study design and participants***

The current study was a double-blind parallel-design randomized controlled clinical trial (RCT) in 66 patients with major burns. The inclusion criteria were 20-45% total body surface area (TBSA) burn, 2^nd^ (deep partial thickness) or 3^rd^ (full thickness) burn degrees and age 18-60 years. Patients with history of diabetes, organ failure, cancer, immune disorders, and allergy or intolerance of protein, body mass index (BMI) < 18.5 kg/m^2^, pregnant as well as, lactating and post-menopausal women, those who needed intensive medical care, enteral or parenteral nutritional support, or those who were reluctant to participate in the study were excluded. All patients received standard medical and surgical care during the study period. The study protocol has been approved by the institutional Ethics Committee of the Iran University of Medical Sciences (Ethics code: IR.IUMS.REC.1397.442) and registered on Iranian registry of clinical trials (IRCT: IRCT20090901002394N42). All patients gave a written informed consent in the beginning of the study.

***2.2 Procedure***

Eligible patients were randomly assigned to either of the three groups of control (n=22), collagen (n=22), or collagen plus fish oil (n=22) based on stratified permuted block randomization using a randomization sequence generated by a random allocation software, with random block sizes of 3 and 6. Patients were stratified based on TBSA < or > 30%. Patients in the collagen group received a drink containing 40 gr hydrolyzed collagen (Rousselot, France) and 10 ml sunflower oil per day, in two divided doses, for three weeks. The combination therapy group received the same amount of collagen in addition to 10 ml/d fish oil (Striver, India), containing 3 gr omega-3 FAs, for three weeks. The control group received a carbohydrate drink with 10 ml/d sunflower oil, similar in taste and color. The drinks were prepared and coded by an independent colleague unaware of the study design. The Toronto formula was used to determine the calorie need of each patient, individually. The protein need was considered 20-25% of total calorie need. 

**2.3 Assessments**

All measurements were performed at baseline, end of weeks two and three. Blood samples were obtained after 8-10 hours of fasting and the sera were kept at -80 °C after centrifugation. Fasting blood glucose (FBG) was determined using enzymatic method (Pars Azmoon Inc., Iran). Fasting insulin levels were measured using enzyme-linked immunosorbent assay (ELISA) kit (Diametra, Italy). HOMA-IR was calculated using the relevant formula: FBG (mg/dL) × fasting insulin (µU/ml) / 405. Hs-CRP concentration was measured based on immunoturbidimetry assay (Roche Diagnostics, Germany). Body weight and height were measured using a scale with height rod (SECA, Germany). A 24-h dietary recall was obtained from all patients by a trained researcher at baseline, and weeks two and three. Dietary reported intakes were analyzed using Nutritionist IV software (N Squared Computing, San Bruno, CA, USA).

**2.4 Statistical analysis**

Based on the relevant formula for RCT studies a sample size of 22 deemed to be adequate to have a power of 80% to detect 30 mg/l difference in hs-CRP levels, when post intervention standard deviation (SD) was assumed to be 33, with a type I error of 0.05 and a drop-out rate of about 15%. The Kolmogorov–Smirnov test was used to assess the normal distribution of variables. Per-protocol analyses were performed to assess differences in the baseline values as well as pre- to post- changes of the outcomes using Chi-Square, analysis of variance (ANOVA), and Kruskal-Wallis tests, where appropriate. The effects of the intervention at the end of weeks two and three were investigated using analysis of covariance (ANCOVA) adjusted for baseline values. Post-hoc analyses were performed to assess the statistical differences between each two groups. Repeated measure and Friedman tests were applied for within group comparisons (pre- to post- changes) in each study group. All analyses were performed using SPSS software (IBM SPSS Statistics for Windows, Version 26.0. Armonk, NY: IBM Corp.). P-value < 0.05 was considered to be statistically significant.

## 3. Results:

**3.1. Baseline characteristics**

In the current study, 66 patients were randomly assigned to three groups to receive either of the collagen, collagen+omega3 or placebo. Three patients, one in each group, did not receive the allocation due to withdrawal or lack of baseline assessments. Three patients in the control (one death, one discharge against medical advice from hospital, one transfer to another hospital), one in the collagen (need for intensive care), and two in the collagen+omega3 (need for intensive care) were excluded from the study. Finally, 18 patients in the control, 20 in the collagen, and 19 in the collagen+omega3 completed the study. 

Baseline characteristics of the study population have been presented in Table 1. There were no significant differences in age, sex, TBSA, burn depth, and burn etiology between the three study groups.

**3.2. Hs-CRP concentration**

**Figure 1:**
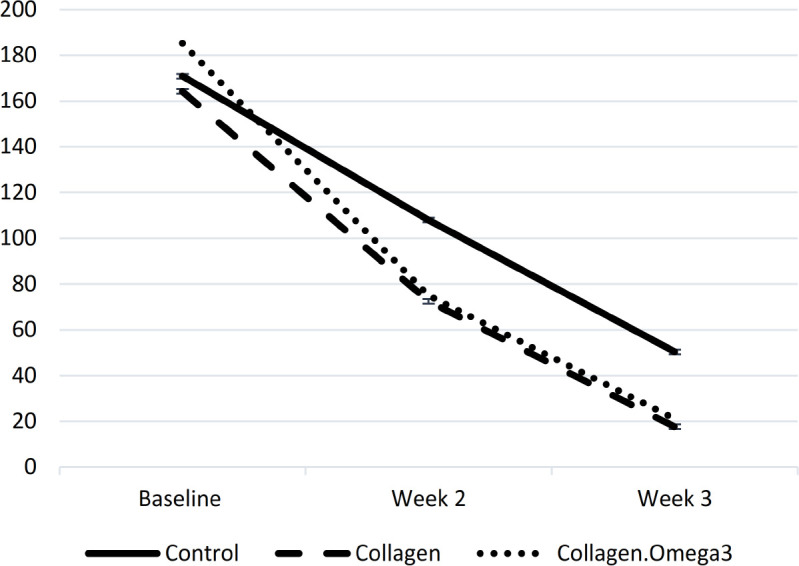
Changes in high-sensitivity C-reactive protein (mg/l) concentration throughout the study period

There was no baseline difference in circulating hs-CRP between the study groups. Following three weeks of the intervention intervention, hs-CRP concentration was significantly different between the study groups (p=0.018) (Table 2). Post-hoc analyses showed considerably lower hs-CRP in the collagen (p=0.026) and collagen+omega-3 (p=0.044) groups compared to the control group. Additionally, pre- to post- (week three) changes of hs-CRP were significantly different between the three study groups (p=0.027). Post-hoc analysis showed significantly higher decrease of hs-CRP only in patients receiving collagen+omega-3, but not collagen alone, compared to the control group (173.2 vs. 103.7 mg/l, p=0.024) (Table 2, Figure 1).

**3.3. Glucose homeostasis**

**Figure 2:**
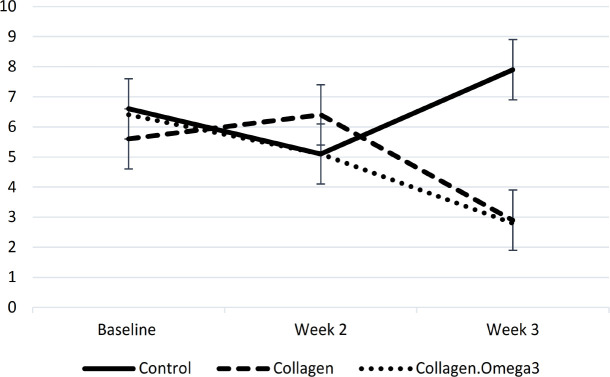
Changes in homeostatic model assessment for insulin resistance values throughout the study period

There were no statistically significant differences in the baseline or post-intervention values of FBG, insulin, and HOMA-IR between the three study groups. There was a significant decrease in FBG levels within the collagen (p=0.002) and collagen+omega-3 (p=0.036) groups at the end of week three compared to the baseline. Additionally, pre- to post- (week three) changes of insulin (p=0.008) and HOMA-IR (p=0.001) were statistically significant only within the collagen+omega-3 group. Post-intervention values of insulin (11.3 and 11.9 vs. 22.8 µIU/ml) and HOMA-IR (2.9 and 2.8 vs. 7.9) seemed to be clinically, but not statistically, lower in both intervention groups compared to the control group (Table 3, Figure 2). In the collagen+omega-3 group, 88.9% of patients had a decreasing trend in HOMA-IR from baseline to the end of the study, while 73.7% of patients in the collagen and 58.8% in the control group had such a trend, and the rest of patients in each group had increased insulin resistance throughout the study.

**3.4. Other variables**

There was no significant difference in body weight at the end of the intervention between the control, collagen, and combination therapy groups (75.8±19, 74.7±12.6, and 73.4±16 kg, respectively, p=0.519). All the study groups had significant improvement in total energy and protein intake compared to their baseline values (all P<0.01). There were no significant differences in energy (2520.1±576.8 vs. 2359.8±605.6 vs. 2449±618.5 kcal/d, p=0.508) or amount of protein intake (121±18.3 vs. 129.7±23.5 vs. 130.6±28.3 gr/d, p=0.545) at the end of the study between control, collagen, and collagen+omega-3 groups, respectively. The percentage of protein intake from energy was significantly higher in the collagen group compared to the control group at weeks two and three, as well as changes in this nutrient on week three compared to baseline (p=0.04, p=0.019, p=0.048, respectively). These differences have been considered in statistical analyses and did not affect the significance of the findings. There were also no significant differences in baseline or post-intervention dietary intakes of carbohydrates, total fats, and micronutrients between the study groups (data not shown). 

## 4. Discussion:

The results of the current trial showed lower hs-CRP levels in patients receiving collagen alone or in combination with omega-3 FAs compared to the control group following three weeks of supplementation; however, the pre- to post- decrease of hs-CRP was significantly higher only in the collagen+omega-3 FAs group compared with the control group. No statistically significant difference was observed in glucose homeostasis between the study groups following the supplementation. However, insulin and HOMA-IR were lower in both intervention groups compared to the control group at week three, which seems to be of clinical importance. The lack of statistically significant differences in secondary outcomes, including insulin and HOMA-IR, between the study groups, might be partly due to the low power of the study; as the sample size has been calculated based on hs-CRP levels as the primary outcome. The reductions in FBG, insulin, and HOMA-IR were statistically significant within the collagen+omega-3 group at week three compared to the baseline. 

Inflammatory markers such as hs-CRP, as well as glucose and insulin levels are recognized to be predictors of survival in burns exceeding 30% TBSA (27). Hs-CRP are increased levels systematically and also locally at tissue levels of the burn site and may be elevated for days and months after burns injury (12, 28). Studies have shown impaired glucose homeostasis, high insulin levels, and insulin resistance for at least 4-5 weeks after the burn (29). Maladaptive responses after injury lead to complications and interfere with the healing process (8, 11, 12). Thus, timely and effective interventions would be of special importance in burns to accelerate alleviation of the metabolic responses. 

No previous study was found investigating the effect of collagen on inflammation and glucose homeostasis in burns or other metabolic stresses. An RCT showed the effect of three months of supplementation with 13 gr/d marine collagen on improvement of FBG, insulin, hemoglobin A1c, hs-CRP, free FAs, and nitric oxide (NO) in patients with type 2 diabetes compared to the control group (30). An experimental study in diabetic mice with full-thickness excisional wound showed significantly lower serum pro-inflammatory cytokines interleukin (IL)-1β and IL-8, higher serum anti-inflammatory cytokine IL-10, as well as better glucose tolerance and fasting glucose following 15 days of supplementation with marine collagen peptide mixture (30 and 45 gr/l) (19). A recent experimental study investigating the effect of eight weeks of marine collagen (100, 200, and 300 mg/kg) supplementation in high-fat fed mice showed decreased insulin concentration and improved HOMA-IR. Collagen supplementation increased the hepatic expression of nuclear factor erythroid 2-related factor 2 (Nrf2) and antioxidant enzymes, superoxide dismutase (SOD) and glutathione peroxidase (GPx), and decreased nuclear factor kappa β (NF-Kβ)-regulated inflammatory enzymes and mediators such as cyclooxygenase-2 and IL-6 (18). Another experimental study showed no significant difference in glucose homeostasis but lower expression of IL-6 and IL-1β in adipocytes following 20 weeks of supplementation with fish collagen (4 g/kg/d) in mice fed a high-fat diet (31). A rat model of diabetes that received marine collagen (2.25, 4.5, and 9 g/kg/d for 4 weeks) indicated improved glucose metabolism and insulin resistance, as well as up-regulated glucose transporter type 4 (GLUT 4) in skeletal muscles, and peroxisome proliferator activated receptor-α (PPAR-α) in the liver. There was a decrease in oxidative stress (higher SOD, GPx, NO, and lower malondialdehyde (MDA)) and inflammation (lower tumor necrosis factor α, CRP, interferon γ) in the intervention compared to the control group. Most effects were observed in medium and high doses of the supplement but CRP levels decreased even with low doses of collagen (20). Collectively, the beneficial effects of collagen on inflammation and metabolic control might be due to its anti-inflammatory, anti-oxidant, and anti-diabetic properties, as well as the specific amino acid content of this protein. Most available studies have used marine sources of collagen and some used large doses of the supplement; however, the current trial showed the beneficial effects of a bovine source of the protein as well. 

Adding omega-3 FAs to collagen was associated with clinical, but not statistical, improvement in insulin resistance and significant decrease of hs-CRP levels compared to the control group throughout the study, which suggests the synergistic effects of collagen and omega-3 FAs. The co-administration of these nutrients, as well as oral intake of omega-3 FAs and the effect of these fats on insulin resistance were rarely investigated in burns so far. Additionally, studies on burns have mostly investigated omega-3 FAs in combination with other nutrients and nutraceuticals, as enteral or parenteral formulas. A double-blind RCT investigating the effect of early enteral feeding enriched with omega-3 FAs (3 gr/d) and glutamine (0.3 gr/kg/d) showed significant decline in CRP levels 7 and 14 days after the injury compared with standard formula in patients with 30-50% TBSA burns (21). A double-blind RCT study has reported a significant decrease in hs-CRP following three weeks of supplementation with isolated soy protein (50 gr/d) alone or in combination with flaxseed oil (30 gr/d) compared to the control group in patients with 20-50% TBSA burn. No additional effect was observed for flaxseed oil on hs-CRP levels in this study (25). An RCT that evaluated the effect of a formula containing fish oil (20% of total fat), arginine (20% of total protein), and glutamine (10% of total protein) compared to standard formula showed no improvement in CRP and immunologic biomarkers in patients with severe trauma or 30-60% TBSA burn (23). Interestingly, a double-blind RCT investigating the effects of a formula supplemented with fish oil (3.5 gr omega-3 FAs/1000 kcal) and arginine (10 gr/1000 kcal) in patients with burns > 20% TBSA showed higher infection and CRP levels in the intervention compared to the control group throughout the study (24). A clinical study in a mixed population with varying degrees of burns, 10-89% TBSA and 0-79% full thickness, age 3-76 years, and length of nutritional support of 7-61 days showed no significant differences in acute phase proteins, CRP, and immunological markers with an enteral formula containing 50% fat from fish oil compared to the control formulas. There was an improving trend of glucose tolerance, indicated by lower need of exogenous insulin (32). The results of this study may be affected by the heterogeneity of the patients’ characteristics. 

Discrepancies in the results of the mentioned studies might be due to differences in their dosage, duration, severity of condition, route of feeding, interactions with other nutrients and nutraceuticals, and the source of omega-3 FAs. 

Omega-3 FAs alter cell membrane fluidity and increase prostaglandin, thromboxane, and leukotriene series with less inflammatory properties compared to eicosanoids derived from arachidonic acid, and decreases inflammatory cytokines (33), which, in turn, could ameliorate the catabolic response. Part of the beneficial effects of omega-3 FAs are conducted by the production of mediators known as “specialized proresolving mediators (SPMs)”. These mediators have a key and active role in altering the principal signs of resolution of inflammation (34, 35). Experimental studies of transgenic models have shown the insulin‐sensitizing, anti-steatotic, and anti-inflammatory effects of SPMs (36, 37), but few clinical studies are available on the subject. 

**Table 1:** Comparing the baseline characteristics of the patients between the three study groups

**Variables**	**Control ** **(n=18)**	**Collagen** **(n=20) **	**Collagen+Omega-3** **(n=19)**	**P**
**Age (year)**				
Mean ± SD	37.3±12.4	33.2±9.6	34±11.4	0.488 ^a^
**Sex**				
Male	14 (77.8)	14 (70.0)	14 (73.7)	0.863 ^b^
Female	4 (22.2)	6 (30.0)	5 (26.3)	
**Burn TBSA (%)**				
Mean ± SD	28.1±9.3	27.5±6.9	29.2±8.4	0.801 ^a^
**Burn depth **				
2^nd^ degree (deep)	18 (100.0)	20 (100.0)	19 (100.0)	1.000
3^rd^ degree	16 (88.9)	17 (85.0)	17 (89.5)	> 0.9 ^c^
**Etiology **				
Flame	13 (72.2)	14 (70.0)	12 (63.2)	0.736 ^c^
Scald	3 (16.7)	4 (20.0)	3 (15.8)	
Alcohol & Thinner	0 (0.0)	1 (5.0)	2 (10.5)	
Electricity	2 (11.1)	1 (5.0)	2 (10.5)	

Values presented as mean ± standard deviation (SD) or frequency (%); a: based on ANOVA test; b: based on Chi-Square test; c: based on Fisher’s Exact test. TBSA: total body surface area.

**Table 2:** High-sensitivity C-reactive protein (Hs-CRP) concentrations in the three study groups before and after supplementation with collagen and omega-3 fatty acids

**Hs-CRP (mg/l)**	**Control** **(n=18)**	**Collagen** **(n=20)**	**Collagen** **+** **Omega-3** **(n=19)**	**P**
**Baseline **	170.9±110.9	164.2±76.2	185.2±83.6	0.746 ^a^
**Week-2 **	108±92.4	72.5±79.9	75±73.1	0.224 ^b^
**Week-3 **	50.4±66.4	17.6±34.1	21.2±30.1	0.018 ^b^
**Week-2 vs. baseline**	62.9±95.6	93.2±85.5	110.3±86.5	0.273 ^a^
P (week-2 vs. baseline) ^c^	0.065	<0.001	<0.001	
**Week-3 vs. baseline**	103.7±90.5	146.6±56.1	173.2±75.7	0.027 ^a^
P (week-3 vs. baseline) ^c^	0.001	<0.001	<0.001	

Values are presented as mean± standard deviation (SD). a: based on ANOVA test; b: based on ANCOVA test adjusted for baseline; c: based on Repeated Measures.

**Table 3:** Glucose homeostasis in the three study groups before and after supplementation with collagen and omega-3 FAs

**Variables**	**Control** **(n=18)**	**Collagen** **(n=20)**	**Collagen+Omega-3** **(n=19)**	**P**
**FBG (mg/dl)**				
Baseline	114.9±27.5	108.5±18.9	114.7±49.2	0.545 ^a^
Week-2	102.4±17	102.2±15.2	101.2±19.1	0.953 ^b^
Week-3	102.8±23.8	95±13.5	93.6±12.2	0.227 ^b^
Week-2 vs. Baseline	12.6±35	6.6±17.6	13.5±46.2	0.539 ^a^
P (week-2 vs. Baseline)^c^	0.091	0.991	0.768	
Week-3 vs. Baseline	12.1±34.1	13.5±17.4	21.1±45.4	0.952 ^a^
P (week-3 vs. Baseline)^c^	0.059	0.002	0.036	
**Insulin (µIU/ml)**				
Baseline	21.1±26.1	17.7±24	22.6±18.9	0.365 ^a^
Week-2	18.8±23.4	25.4±23.7	18.6±17.4	0.593 ^b^
Week-3	22.8±45.6	11.3±11.2	11.9±13.4	0.388 ^b^
Week-2 vs. Baseline	3±34.7	-7.5±36	4±28.4	0.178 ^a^
P (week-2 vs. Baseline)^c^	0.523	0.51	1	
Week-3 vs. Baseline	-1.1±54.6	7.1±24.2	10.3±25.8	0.283 ^a^
P (week-3 vs. Baseline)^c^	0.523	0.51	0.008	
**HOMA-IR**				
Baseline	6.6±8.5	5.6±9.9	6.4±5.2	0.436 ^a^
Week-2	5.1±7.0	6.4±6.2	5.1±5.7	0.798 ^b^
Week-3	7.9±20.7	2.9±3.1	2.8±3.2	0.356 ^b^
Week-2 vs. Baseline	1.7±11.4	-0.7±12.8	1.3±8.2	0.156 ^a^
P (week-2 vs. Baseline)^c^	0.381	0.51	1	
Week-3 vs. Baseline	-1.1±23.1	3±10.1	3.4±6.6	0.292 ^a^
P (week-3 vs. Baseline)^c^	0.309	0.51	0.001	

Values presented as mean ± standard deviation (SD). a: based on kruskal-wallis test; b: based on ANCOVA test adjusted for baseline; c: based on Friedman test. FBG: fasting blood glucose; HOMA-IR: homeostatic model assessment for insulin resistance.

## 5. Limitations

This study had some limitations including the lack of oral glucose tolerance test, which could have made the results more precise. Additionally, investigating other indicators of inflammation as well as the plausible mechanisms of the observed changes should be considered in future studies with larger sample sizes. Part of the non-statistically significant, although probably clinically important, differences of post-intervention glucose homeostasis parameters in this study might be due to the relatively low sample size for these secondary outcomes.

## 6. Conclusion

Supplementation with collagen hydrolysate and omega-3 FAs improved hs-CRP concentration and insulin resistance in patients with major burns, although the latter did not reach statistical significance. Omega-3 FAs had additional effects on modulating inflammation. Further large and well-designed clinical trials are required to confirm the current findings, especially in terms of glucose homeostasis, in patients with major burns.

## 7. Declarations

### 7.1. Acknowledgment

None

### 7.2. Author contribution

Conceptualization and design, E.A., M.J.H.A., M.D., M.Y., S.M.R. and Sh.J.; methodology, E.A., M.J.H.A., M.Y. and Sh.J.; formal analysis, E.A. and M.Y.; investigation, E.A., Sh.S., M.R.E. and M.H.; data curation, E.A., Sh.S., M.R.E., M.H. and M.Y.; writing the original draft, E.A.; writing review and editing, E.A., M.J.H.A., M.D., M.Y., S.M.R. and Sh.J.; supervision, M.J.H.A., M.D., S.M.R. and Sh.J.; funding acquisition, M.J.H.A. and Sh.J. All authors have read and agreed on the published version of the manuscript.

### 7.3. Funding

This study has been supported by Iran University of Medical Sciences grants 33869 and 12325, and Tehran University of Medical Sciences grant 43535.

### 7.4. Conflict of interest

None
